# Bispectral Index Guidance Reduced Target Plasma Propofol Concentration During ERCP in Patients with Liver Cirrhosis

**DOI:** 10.4274/TJAR.2024.241635

**Published:** 2024-10-30

**Authors:** Yasmin Kamel, Noura Sasa, Madiha Naguib, Khaled Ahmed Yassen, Eman Sayed

**Affiliations:** 1 National Liver Institute, Menoufia University, Department of Anaesthesia, Shebeen El-Kom, Egypt; 2 National Liver Institute, Menoufia University, Department of Internal Medicine, Shebeen El-Kom, Egypt; 3 King Faisal University College of Medicine, Anaesthesia Unit/Department of Surgery, Alahsa, Saudi Arabia

**Keywords:** Cholangiopancreatography, endoscopic retrograde, liver cirrhosis, prone position, propofol, syringe pumps

## Abstract

**Objective:**

The primary aim of this study was to investigate the guidance effect of the bispectral index (BIS) on the target plasma concentration (TPC) of propofol required for deep sedation during endoscopic retrograde cholangiopancreatography (ERCP). Second, to identify propofol consumption, recovery time, and adverse events.

**Methods:**

A total of 42 consecutive patients with liver cirrhosis and 43 consecutive patients with healthy livers were enrolled. Propofol was administered via a target control infusion (TCI) syringe pump (Marsh Model) at BIS 60-70. Patients were not intubated, were placed in the prone position, and underwent spontaneous breathing. Propofol TPCs (µg mL^-1^) and BIS values were recorded at T0 (baseline), T1 (5 min after induction), T2 (5 min into ERCP), T3 (15 min), T4 (30 min), and T5 (recovery).

**Results:**

TPCs and propofol consumption were lower in patients with cirrhosis than in those without cirrhosis (T4: 2.7±0.5 vs. 3.3±0.4 µg mL^-1^), *P*=0.001, and 270.4±6.9 mg vs. 390.8±13.4 mg, *P*=0.001), respectively. Patients with cirrhosis required more time to recover (8.5±2 vs. 6.2±0.9 min, *P*=0.001), despite comparable ERCP durations (31.1±11.1 vs. 34±12.5 min, *P*=0.28). A significant decline in TPC values among patients with cirrhosis with time (T1: 3.3±0.3, T2: 3.1±0.3, T3: 2.9±0.4, T4: 2.7±0.5 µg mL^-1^, *P*=0.001), indicating a cumulative effect. One patient with cirrhosis required bag-mask ventilation, while three patients without cirrhosis were converted to general anaesthesia.

**Conclusion:**

Combining the TCI Marsh pharmacokinetic model with BIS monitoring lowered the TPC levels required for deep sedation in patients with cirrhosis compared with healthy patients and allowed for individual variations. The prone position in deeply sedated and non-intubated spontaneous breathing patients is not without the risk of hypoxia.

**Main Points**

• Bispectral index-guided sedation toward optimal and lower target plasma propofol concentrations in liver cirrhotic patients compared to healthy counterparts.

• Prone position in patients undergoing spontaneous breathing is not without risk of hypoxia.

• Attention should be paid to the development of hypoxia and desaturation throughout the procedure, and the presence of a qualified anaesthesiologist at these remote endoscopy sites is essential.

## Introduction

Endoscopic retrograde cholangiopancreatography (ERCP) has recently been performed with deep sedation more than with general anaesthesia (GA).^1^ In 2021, ERCP under GA only ranged from 7% to 10% in the United Kingdom.^2^ Deep propofol sedation for ERCP procedures is preferred to conscious sedation as patients often tolerate the procedure better.^2, 3^ However, deep sedation, as defined by the American Society of Anesthesiologists (ASA), can lead to airway compromise and inadequate spontaneous breathing. Propofol has a narrow therapeutic window, and patients can progress from deep sedation to GA. Individual variations and co-existing diseases, such as liver cirrhosis, should be taken into consideration.^4, 5^ The Royal College of Anaesthetists in the United Kingdom recommends that patients undergoing deep sedation should be monitored and an anaesthesiologist should be present.^6^ Target control infusion (TCI) syringe pumps are designed to deliver propofol at a specific target plasma concentration (TPC) for sedation or anaesthesia, which can range from 2 to 5 µg mL^-1^. However, the pharmacokinetic models incorporated in these TCI syringes were derived from pharmacological studies performed among patients without hepatic disorders, which might not be suitable for patients with hepatic cirrhosis.^7, 8, 9, 10, 11^ Hepatic disease can affect drug pharmacokinetics and dynamics.^12, 13, 14, 15^ Inadequate sedative doses to hepatic patients can delay recovery and lead to drug accumulation.^13, 16^ The primary aim was to investigate the guidance effect of monitoring sedation depth with the bispectral index (BIS), an electroencephalogram (EEG)-processed monitor, on the required TPC of propofol required for deep sedating patients with and without hepatic cirrhosis during ERCP. Secondary to identify propofol consumption and recovery time, as well as reporting any adverse events associated with deep sedation.

## Methods

The Institution Review Board of National Liver Institute, Menoufia University authorized (IRB NLI IRB 00003413 FWA0000227) this quasi-experimental study on the 1^st^ of November 2019, with approval number 0177/2019. The study was conducted between 10^th^ November 2019 and 1^st^ November 2021 at National Liver Institute, Menoufia University, Egypt. All patients in the study provided informed consent to participate.

### Inclusion Criteria

Patients aged 18-60 years who underwent elective ERCP. Patients were not intubated, were placed in a prone position, and underwent spontaneous breathing. Two groups of patients: the cirrhotic group that included forty-two consecutive hepatic cirrhotic patients with Child-Pugh classification (Child A or B) and with confirmed laboratory and ultrasound diagnosis for hepatic cirrhosis from chronic hepatitis C, which represent the main etiology of cirrhosis in this part of the world.^17^ The non-cirrhotic group included 43 consecutive patients with healthy livers. Two patients were excluded from the cirrhotic group and three from the noncirrhotic group.

### Exclusion Criteria

Participants with a history of severe chronic obstructive lung disease and a significant risk of aspiration. Patients were also excluded if they faced procedural or anatomical challenges not related to the sedation technique that could prolong the duration of the ERCP, when converted to GA with tracheal intubation, or if the procedure was aborted. In the study by Fanti et al.,^18^ difficult ERCP affected the total dose of propofol consumed and the mean duration of ERCP. Both were related to the degree of procedural difficulty.^18^ The exclusion criteria include patients with significant hepatic encephalopathy or coma, cardiovascular, respiratory, or renal diseases, drug abuse, and morbid obesity. Patients with significant encephalopathy have abnormal EEG results, which can affect the EEG and hence the BIS values, as stated by Mitra et al.^19^. The severity of hepatic encephalopathy was assessed using the West Haven criteria on a scale of 0-4. Stages 0-1 are minimal hepatic encephalopathy in which symptoms may not be noticeable clinically and were included in the study. Stage 2-4 is characterized by an increase in severity, and stage 4 is in coma.^20, 21^

### Target Control Infusion Technique

The TCI technique ensures that propofol reaches and maintains a desired concentration in the blood or at the effect site (Brain) via computerized syringe pumps, which constantly alter the propofol dosage. TCIp indicates that the blood plasma concentration for the drug is the principal target, whereas the target in TCIe is the effect site (Brain) concentration. In the current study, the TCIp was adopted, and doses were altered according to the changes in the BIS to keep it between 60 and 70. TCI models are based on pharmacokinetic studies embedded in the software of the smart syringe pump. For propofol, the Marsh and Schnider models are widely available; however, a newly developed model called the Eleveld model was recently introduced. The Marsh model was adopted in the current study.^22, 23^

### Deep Sedation Technique and Monitors

The ASA classified levels of sedation as minimal, moderate (conscious), or finally deep sedation, which can easily drift into GA. ERCP can be performed under moderate conscious sedation, with midazolam and opioid or under deep sedation with propofol.^24, 25^ In the current study, the anaesthesiologists provided deep sedation with monitored care to the patients, which was in line with Azimaraghi et al.^26^ consensus for sedation. Azimaraghi et al.^26^ favored monitoring deep sedation care over GA during ERCP, but with specific inclusion and exclusion criteria to reduce perioperative adverse events, and the criteria were respected in the current study. Patients with an increased risk of pulmonary aspiration and those undergoing prolonged high-complexity or difficult procedures were not considered for deep sedation and were excluded.^26^

The current study protocol does not allow premedication for any patient before induction. In the endoscopy suite, standard monitors [General Electric (Madison, USA)] were applied, including non-invasive blood pressure (NIBP), electrocardiogram (ECG), pulse oximetry with oxygen saturation (SaO_2_), and end-tidal carbon dioxide percentage (ETCO_2_) sampled from a modified nasal cannula capable of simultaneously delivering oxygen and sampling carbon dioxide (CO_2_) at the same time. End-tidal carbon dioxide monitored the breathing rhythm and allowed early warning for any episodes of apnea, besides visual monitoring of chest movements. Qadeer et al.^27^ demonstrated that hypoxia was reduced during ERCP by continuous monitoring of end-tidal CO_2_ during the procedure. The wrist or forearm vein of the independent arm was cannulated for intravenous fluid and propofol infusion. Before sedation, each patient was independently positioned to avoid any possible nerve injury from passive positioning. Ringer’s acetate (500 mL) was infused before commencing endoscopy. A 50 mL syringe containing 10 mg mL^-1^ propofol (Fresenius Kabi, Bad Homburg, Germany) was loaded into an automated, computer-controlled syringe pump (Agilia, Fresenius Kabi, Germany), and the Marsh pharmacokinetic model was selected. Age and weight were also added to the settings. The initial TPC was set at 4 µg mL^-1^. After administration of 100% oxygen via the nasal cannula, the propofol TPC and doses were titrated to keep the patients deeply sedated at a BIS value (BIS, Aspect, MA, USA) between 60 and 70. BIS monitoring facilitates objective assessment of the sedation level during the procedure.^28^ Recovery was defined as recovery after restoring consciousness or BIS values increase above 90. BIS monitoring is an EEG-processed method that guides the depth of anaesthesia using a complex algorithm to create an index score. BIS objectively measures the level of consciousness as mentioned above and titrates the propofol dosage toward the desired effect. Any increase in BIS readings >70 indicates the inadequacy of sedation and the need to increase the targeted plasma concentration in steps of 0.5 µg mL^-1^ every 20 seconds and vice versa until BIS falls back to values between 60 and 70.^29^

### Precautions During Deep Propofol Sedation

Propofol is a short-acting intravenous anaesthetic agent with better sedation and recovery outcome compared to conscious sedation.^30^ However, Propofol has a narrow therapeutic window, and it can easily progress from deep sedation to GA, which can affect airway patency and spontaneous breathing. The presence of an anaesthesiologist and continuous monitoring of breathing, SaO_2_, and ETCO_2_ are mandatory to allow for early air way obstruction warning.^31^ The Royal College of Anaesthetists in the United Kingdom recommends that the presence of an airway supporter to immediately interfere when in need.^32^ Oxygenation is maintained during spontaneous breathing with 100% oxygen at 4-8 L min. Airway opening skills, such as jaw thrust and head tilt and chin lift, should be applied initially to relieve obstruction whenever SaO_2_ falls below 90% or the capnography waves become interrupted. However, if this approach is insufficient, the patient should be moved to the prone position for manual ventilation and endotracheal intubation if necessary.

### Maintenance of Hemodynamics

Hypotension is defined as a reduction of >20% of the baseline mean NIBP. Hypotension should be initially assessed for hypovolemia, and fluids should be replaced when required. Otherwise, treatment with intravenous boluses of ephedrine (5 mg). Bradycardia [heart rate (HR) <45 beats min] should be treated with Atropine (0.25 mg). Any increase in HR (beat min) or mean MAP mm Hg by more than 20% of baseline within a BIS value between 60 and 70 indicates the need for fentanyl. Adverse events, such as hypoxia, hypotension, and bradycardia were all recorded.

### Data and Measured Times

HR (beat min), mean NIBP (mmHg), SaO_2_ (%), TPC (µg L^-1^), BIS values at T0 (baseline), T1 (5 minutes after induction), T2 (5 min ERCP), T3 (15 min ERCP), T4 (30 min ERCP), and T5 (end ERCP).

### Power of the Study

The power was achieved by a sample size of 40 patients per group (number of groups is 2) for the t-test means: difference between to independent means (two groups) based on a comparison of total anaesthetic consumption (primary outcome), resulting in a two-tails standardized effect size (d) of 1.160 and a power of 99.92%. A sample size of 40 patients per group is sufficient to conduct this study with a power of >80%. The post-hoc computation for the achieved power was performed using G^*^Power version 3.1.9.2.^33^

### Statistical Analysis

Demographics, monitor parameters and TPC data were expressed as mean and standard deviation for analysis. Data were loaded into the Statistical Package for Social Science (SPSS) software package (version 21) (SPSS, Inc., Chicago, IL, USA). Repeated measures ANOVA (chi-square) was applied between the measured times. Propofol TPC and SaO_2_ in the studied groups are shown as clustered bar charts with a 95% confidence interval (Dunn-Sidek technique). T-test for comparisons performed between the two groups.

## Results

**Figure 1:**
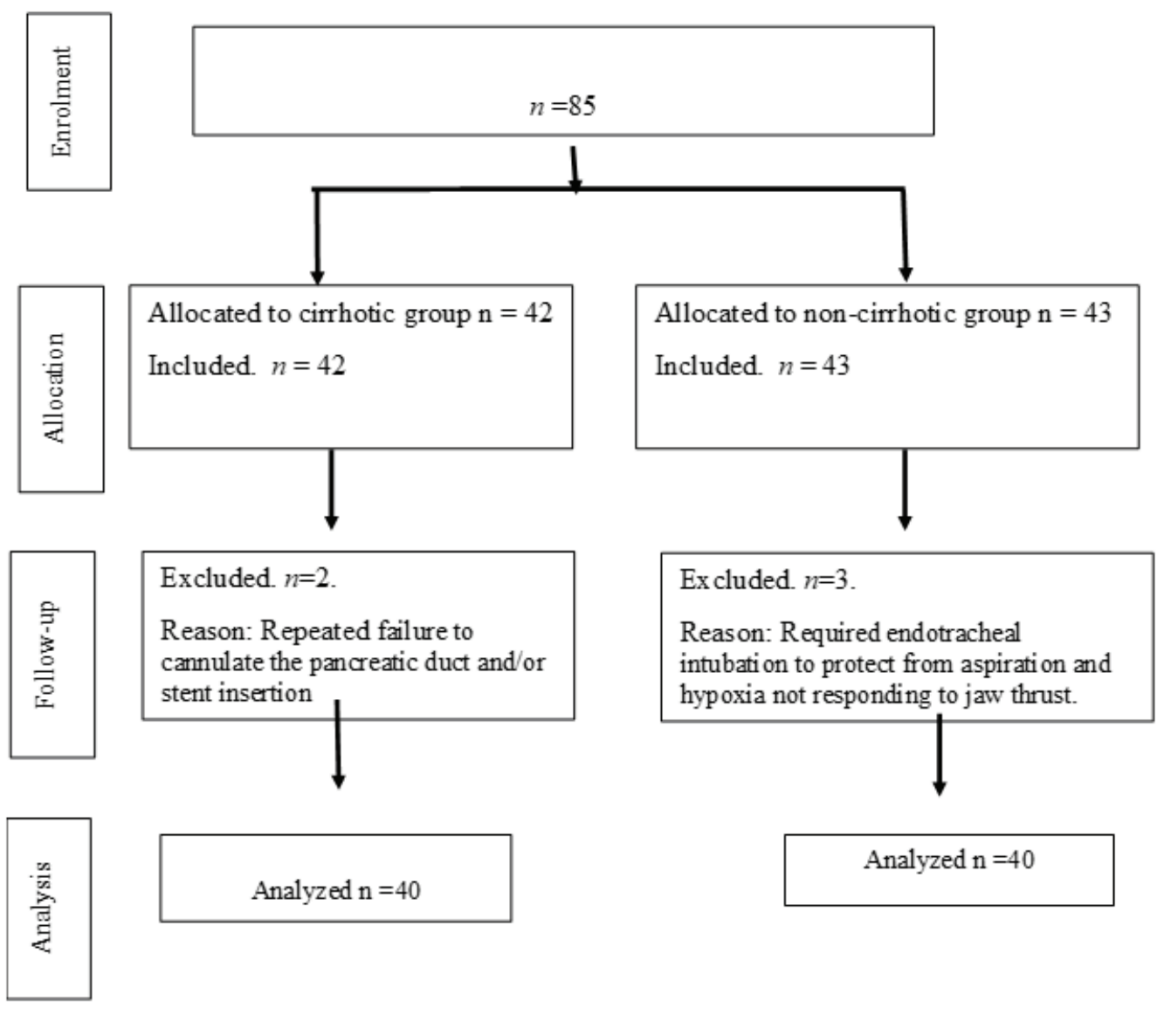
Consort flow graph

Eighty-five patients were enrolled in this study. Only five participants were excluded, as demonstrated in the CONSORT flow chart in Figure 1. Forty-two consecutive patients were allocated to the cirrhotic group, and 43 consecutive patients with healthy livers were allocated to the noncirrhotic group. Two patients were excluded from the cirrhotic group and three from the. Table 1 presents the demographic characteristics of the included patients in each group. Age 47.93±11.62 vs. 47.43±10.62-years, *P*=0.84, and body mass index 26.89±2.58 vs. 27.15±2.91 kg m^2-1^, *P=*0.67 in cirrhotic versus non-cirrhotic patients, respectively. None of the included patients had significant neurological disorders. Hypertension was the most frequent cardiovascular comorbidity (30%), and 10% had a previous history of biliopancreatic surgery.

**Figure 2:**
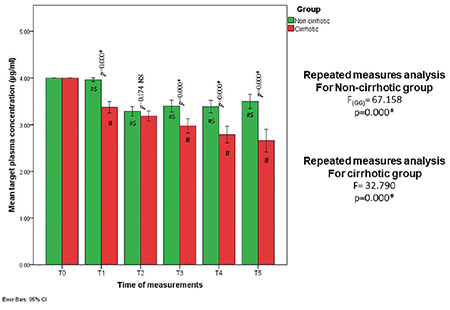
Target plasma concentration in the studied groups, shown as a clustered bar chart with a 95% confidence interval (Dunn-Sidek technique). T0 (baseline), T1 (5 min after induction), T2 (5 min ERCP), T3 (15 min ERCP), T4 (30 min ERCP), and T5 (End ERCP) ERCP, endoscopic retrograde cholangiopancreatography; #means statistical significance with measurement time T0, $means statistical significance with previous time of measurement.

**Figure 3:**
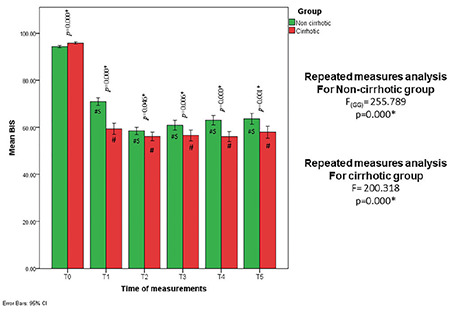
Mean BIS in the studied groups, shown as a clustered bar chart with a 95% confidence interval (Dunn-Sidek technique). T0 (baseline), T1 (5 min after induction), T2 (5 min ERCP), T3 (15 min ERCP), T4 (30 min ERCP), and T5 (End ERCP) BIS, bispectral index; ERCP, endoscopic retrograde cholangiopancreatography; #means statistical significance with measurement time T0, $means statistical significance with previous time of measurement.

Hepatic patients (Child-Pugh classification: A 50% and B 50%) consumed less total propofol for sedation during ERCP (270.48±6.91 mg vs.390.88±13.44 mg, *P*=0.001), (Table 1). A lower propofol TPC was required to sedate patients with cirrhosis compared with patients without cirrhosis (T4: 2.7±0.5 vs. 3.3±0.4 µg mL^-1^) (*P*=0.001). Total propofol consumption and TPC were significantly reduced among patients with cirrhosis compared with those without cirrhosis when guided by BIS. The mean recovery times (minute) were longer among cirrhotic vs. non-cirrhotic patients (8.53±2.09 vs. 6.25±0.90; *P *< 0.001, respectively), despite similar ERCP durations (Table 1 and Figure 2). The mean BIS values for patients with cirrhosis tend to drift to lower values (BIS<60) compared with those with healthy livers (T1: 59.40±7.30 vs 70.95±5.13; *P* < 0.001, T2: 56.13±5.76 Vs 58.50±4.67; *P*=0.05, T3: 56.58±7.32 vs 60.98±6.50; *P*=0.006, T4: 56.08±6.42 vs 63.08±6.30; *P *< 0.001, T5: 58.00±6.61 vs 63.63±6.92; *P*=0.001, respectively), as shown in Table 2 and Figure 3.

Another significant finding was the gradual decrease in the BIS-guided propofol TPC (µg mL^-1^) required to deeply sedate patients with cirrhosis as time proceeds with ERCP, suggesting a cumulative effect: T1: 3.3±0.3, T2: 3.1±0.3, T3: 2.9±0.4, T4: 2.7±0.5, repeated-measures ANOVA, *P*=0.001. Figure 2. The systemic hemodynamics were not different between the two study groups (*P* > 0.05) (Table 3). No intraoperative awareness was reported for any of the study patients.

In the cirrhotic group, only one patient required temporary bag-mask ventilation to support his breathing, and ERCP was resumed immediately. Three non-cirrhotic patients required endotracheal intubation to treat desaturation and avoid aspiration during prolonged ERCP and were excluded from the study (Figure 1).

## Discussion

The optimal propofol TPC for deep sedation when guided by BIS was found to be lower for patients with cirrhotic livers compared with those with healthy livers, as shown in the results. Liver cirrhosis leads to a reduction in liver mass and hepatic blood flow, which can affect propofol pharmacokinetics, dynamics, and clearance.

### Pros of Processed ECG Monitoring

One of the lessons learned from the current study is the ability of the BIS to identify individual variations. The TPC of propofol for deep sedation was gradually and progressively reduced among patients with cirrhosis, specifically as the ERCP progressed from one measurement time to another, indicating a cumulative effect. These findings support the beneficial role of the BIS as a processed EEG monitor for sedation depth and as a guide for the optimal propofol TPC. These findings agree with the recommendations and guidelines for safe practice published by the Association of Anesthetists and the Society for Intravenous Anesthesia in 2019,^34^ as well as those extracted from the work by Castellanos Peñaranda et al.^35^.

Few publications have investigated the impact of monitoring the depth of sedation on the consumption of hypnotic medications in this specific group of patients with liver cirrhosis. Deep sedation can easily drift into GA (<BIS 60), particularly among hepatic patients, as evident from the mean BIS values compared with the controls (Table 2), which warrant the need for continuous monitoring of the BIS values and frequent adjustment of the propofol infusion rates to prevent any further increase in sedation depth. However, few patients in both groups required assisted breathing and endotracheal intubation. This study demonstrated the importance of combining BIS monitoring with TCI. Manual propofol injection or continuous infusion without EEG monitoring or TCI software is not recommended. There is a need to train anaesthesiology staff on TCI protocols for sedation and explain the beneficial role of monitoring sedation depth using processed EEG monitors on a wider scale, as recommended by the Total Intravenous Association.

Entropy, another processed EEG monitor, also revealed similar findings to GA in surgery when applied to hepatic patients, as in Yassen et al.^36^, Vakkuri et al.^37^, and Wang et al.^38^ studies. Schumann et al.^39^ Yassen et al.^40^ and Refaat and Yassein^41^ believe that anaesthesia depth monitors should be implemented and encouraged. This will help identify variations in individual responses to different anaesthetic agents. Processed EEG monitors should be combined with other standard monitors to enable a multimodal monitoring approach. In the current trial, the dual monitoring of the BIS and other hemodynamic parameters helped reduce drug delivery and hemodynamic instability. Sessler et al.^42^ their study showed that low BIS levels were correlated with both low mean blood pressure and minimum alveolar concentrations. They linked this to increased hospitalization and mortality. Leslie et al.^43^ reported a relationship between low BIS values and survival.

### Limitations of Processed EEG Monitoring

Processed EEG monitors are not without limitations and practical challenges. Hajat et al.^44^ review in 2017 discussed the limitations raised by the National Institute for Clinical Excellence (NICE) in 2013. The NICE report supports their use, particularly in patients at higher risk. However, evidence of their impact on reducing awareness is not enough.^45^ Ibrahim et al.^46^ noted that BIS scores can vary significantly between patients, making it difficult to predict ED depth without considering individual variations. In the current study, the results support these allegations. BIS values not only varied from one patient to another but also from a measured time to another in the same patient. One of the arguments that limit the spread of processed EEG monitoring among anaesthesiologists is the belief that monitoring end-tidal concentrations of inhaled anaesthetics can represent an accurate reflection of the drug’s effect on the brain. However, these end-tidal concentrations will never reflect individual variations. The cost and availability of EEG depth monitors worldwide remain challenges. Most processed EEG devices derive their results from sampling the frontal area, not the rest of the brain.

Recently during the Euroanesthesia 2024 Meeting; May 25-27; 2024; in Munich, Germany, Matthias Kreuzer, from the Technische Universität München, Germany, discussed how hypotension, hypoxia, hypercarbia, and the combination of more than one anaesthetic drug could affect EEG interpretation.^47^ In our study, no hypotensive events were reported, and only propofol was infused. Recently, in 2020, Kaiser et al.^48^ conducted a narrative review discussing the pros and cons of the available EEG monitors and the need to respect individual variations, particularly among the elderly.

### Marsh Target Control Infusion Model

The Marsh pharmacokinetic parameters incorporated into the TCI smart syringes, as previously mentioned, were designed for patients without organ dysfunction and might not be optimal for patients with hepatic disease. Wu et al.^49^ measured propofol plasma concentrations and discovered significant changes during the three stages of liver transplantation. The preset TCI model does not take into consideration these significant changes in propofol plasma concentrations, and a method is required to guide propofol doses. Tremelot et al.,^50^ later in 2008 confirmed these propofol pharmacokinetic changes during the anhepatic phase of liver transplantation. Tremelot et al.^50^ had to decrease the propofol TPC during the anhepatic phase to 2.0 µg mL^-1^ ±0.8 compared to 3.0 µg mL^-1^ ±0.9, (*P* < 0.0001) in the other phases of the transplant procedure. The above two studies indicate that liver patients should not be subjected to the same TCI Marsh pharmacokinetic settings as for other patients with healthy livers and that a method to monitor the effect of the drug should be introduced to guide the TCI settings. Joosten et al.^51^ in 2020 developed a multiple closed-loop system that included a TCI syringe pump and a BIS monitor together with a carbon monoxide monitor (Flotrac, Edwards Life sciences, USA). This system was able to provide promising results but needed to be evaluated in large populations. Kamel et al.^52^ studied a group of patients with cirrhosis undergoing liver resection using the Marsh model and found that an adequate TPC for propofol with fentanyl was 3.00 µg dL^-1^.

### Accuracy of Target Control Infusion Models

TCI models were created from studies performed on a limited group of patients, and thus, they might not accurately represent the vast variety of patients encountered in daily practice. A pharmacokinetic model based on a wider population is still needed to reflect and describe adequate plasma concentration changes and predicted plasma concentrations. The effect site brain concentration might not improve the performance of the current pharmacokinetic (PK) models, but adopting more improved PK models will. The Eleveld propofol model is one of these recently developed models, which is considered to be more accurate in predicting plasma concentrations and more applicable to a wider range of patients than the Marsh and Schnider models. However, the Eleveld model needs to be installed on a wider scale, and more PK models need to be designed to target specific patient populations, such as patients with liver dysfunction and cirrhosis.^53^

### Hypoxia and Desaturation

The main challenge in the current study was the remote position of the endoscopy suite, which should be equipped with the same standard facilities, such as those prescribed by the ASA in 2018, for operating rooms. The procedures include the presence of a qualified anaesthesiologist and anaesthesia machines with electrocardiogram, NIBP, SaO_2_, and capnography. The American Society for Gastrointestinal Endoscopy also published guidelines for procedural sedation, which are similar to the ASA recommendations; but unfortunately, the capnography monitoring was not considered mandatory.^54, 55, 56, 57^

Sedation-related complications prescribed by Azimaraghi et al.^26^ and Hormati et al.^58^ include desaturation and pulmonary aspiration, as well as hemodynamic instability and apnoea.

Hypoxia can develop with deep sedation Metzner et al.^59^ and Goudra et al.^60^ reported that desaturation can double that of operating rooms. Goudra et al.^61^ in a retrospective analysis showed that 72% of the adverse events in the endoscopic setting were related to desaturation.

One of the lessons learned from the current study was the need to monitor the capnography rhythm and chest movements continuously and interfere when needed. Four of the enrolled patients required interference to protect them from desaturation, as mentioned in the results section. The availability of an anaesthesiologist to manage airway obstruction was recommended by the European Society of Anesthesiology and in the European Board of Anesthesiology guidelines for procedural sedation and analgesia in adults.^62^

Hypoxia in patients with prone-positioned spontaneous breathing represents a serious adverse event. Melis et al.^63^ study a significant portion of their patients suffered from desaturation (35%); they were non-intubated healthy persons prone to undergoing ERCP with TCI propofol. In the current study, the results indicated that three patients developed hypoxia (3/85, 3.5%) and were intubated and excluded from the study. One patient required temporary supportive facemask ventilation during ERCP, and the procedure was not aborted. Smith et al.^30^ conducted a randomized control trial and reported a 10% conversion rate to GA in high-risk patients, but this rate was significantly reduced with lower ASA grades (1 or 2) to 3.7%, which is similar to the 3.5% in our current study.^64^

The recovery time was statistically prolonged among patients with cirrhosis compared with the controls, but without noticeable clinical significance; however, in a high-turn flow endoscopy unit, this could have an impact on the ready-to-discharge time, which unfortunately was not studied and can be considered one of the limitations in the study. However, given the cumulative effect observed with TPC among the cirrhotic patient group only, one would expect a significant delay in hepatic recovery would be expected if TPC were not monitored and guided with BIS.

Finally, Hormati et al.^58^, Althoff et al.^2^ and Khoi et al.^65^ and reported an increase in hypotensive events with GA during ERCP compared with deep sedated with propofol. Fortunately, hypotension was not observed in the current study. This could be due to the careful selection of the included patients or the combination of TCI with BIS monitoring, which helped to avoid overdosing and to respect individual variations.

## Conclusion

In conclusion, combining the Marsh TCI pharmacokinetic model with BIS monitoring reduced the TPC required for deeply sedating patients with cirrhosis undergoing ERCP and identified individual variations. This study demonstrated the importance of shifting to TCI during deep sedation and avoiding manually injecting propofol or continuously infusing propofol with ordinary syringe pumps without a mean of sedation depth monitoring, particularly among patients with hepatic cirrhosis. The prone position in patients without intubated spontaneous breathing is not without risk. Attention should be paid to hypoxia and desaturation development throughout the procedure. Adhering to the exclusion criteria, monitoring of breathing and the presence of a qualified anaesthesiologist at these remote endoscopy sites are essential.

## Figures and Tables

**Table 1. Demographics and Perioperative Study Findings:** 

	**Group**		**Test of significance** ***P *value**
	**Non-cirrhotic**	**Cirrhotics**	
**Age (year)** **n** **Mean ± SD**	40 47.43±10.67	40 47.93±11.62	t_(df=78)_ = 1.09 *P*=0.28, NS
**BMI (kg m²)** **n** **Mean ± SD**	40 27.15±2.91	40 26.89±2.58	t_(df=78)_ = 0.43 *P*=0.67, NS
**Total procedure time (min)** **n** **Mean ± SD**	40 47.43±10.67	40 31.15±11.15	t_(df=78)_ = 1.09 *P*=0.28, NS
**Total propofol (200 mg/20 mL) consumption (mg)** **n** **Mean ± SD**	40 390.88±13.44	40 270.48±6.91	t_(df=78)_ = 5.19 *P*=0.000*
**Recovery time (min)** **n** **Mean ± SD**	40 6.25±0.90	40 8.53±2.09	t_(df=78)_ = 6.33 *P*=0.000*

Age expressed as years and body mass index (BMI) expressed as kg m^2^.

*Denotes statistical significance (p<0.05), while NS denotes statistical non-significance (*P* > 0.05).

SD, standard deviation; t, independent Student’s t-test; df, degree of freedom; n, number of patients.

**Table 2. Bispectral Index (BIS) Trend Changes in the Two Studied Groups:** 

**BIS**	**Group**		**Test of significance** ***P *value**
	**Non-cirrhotic**	**Cirrhotics**	
**T0** **n** **Mean ± SD**	40 94.40±1.35	40 95.90±1.46	t_(df=78)_ = 4.76 *P*=0.000*
**T1** **n** **Mean ± SD**	40 70.95±5.13	40 59.40^#^±7.30	t_(df=69.98)_ = 8.18 *P*=0.000*
**T2** **n** **Mean ± SD**	40 58.50±4.67	40 56.13^#^±5.76	t_(df=78)_ = 2.03, *P*=0.05*
**T3** **n** **Mean ± SD**	40 60.98±6.50	40 56.58^#^±7.32	t_(df=78)_ = 2.84, *P*=0.006*
**T4** **n** **Mean ± SD**	40 63.08^#$^±6.30	38 56.08^#^±6.42	t_(df=76)_ = 4.855 *P*=0.000*
**T5** **n** **Mean ± SD**	38 63.63^#$^±6.92	28 58.00^#^±6.61	t_(df=64)_ = 3.3, *P*=0.001*
**Repeated measures ANOVA** **Chi-square** **p**	F_(GG)_= 255.789 *P*=0.000*	F= 200.318 *P*=0.000*	

T0 (baseline), T1 (5 minutes after induction), T2 (5 min ERCP), T3 (15 min ERCP), T4 (30 min ERCP), and T5 (End ERCP).

*Denotes statistical significance (*P* < 0.05), while NS denotes statistical non-significance (*P* > 0.05). ^#^Means statistical significance with measurement time T0, ^$^means statistical significance with previous time of measurement.

SD, standard deviation; t, independent Student’s t-test; df, degree of freedom; n, number of patients.

**Table 3. Systemic Hemodynamics of Patients:** 

**Variables**	**Mean ± SD**		**Test of significance** ***P *value**
	**Non-cirrhotic**	**Cirrhotics**	
**Heart rate (beat min)** **T0**	80.53±9.64	90.00±14.21	t_(df=78)_ =3.49 *P*=0.001*
**T1**	77.53**^#^**±10.43	89.18±13.76	t_(df=78)_ =4.27 *P*=0.000*
**T2**	79.93**^#^**±9.82	88.20±14.25	t_(df=69.232)_ =3.03, *P*=0.003*
**T3**	82.55**^#^**±9.37	89.35^#^±12.50	t_(df=72.33)_ =2.75 *P*=0.007*
**T4**	85.92**^#^**±10.13	88.54^#^±12.76	t_(df=76)_ =1.00, *P*=0.319 NS
**T5**	85.37**^#^**±9.18	88.73^#^±12.31	t_(df=69)_ =1.31 *P*=0.19 NS
**Repeated measures ANOVA (chi-square)** ***P***	f_(GG)_=12.364 P=0.000*	f_(GG)_ = 0.559, *P*=0.662 NS	
**Mean blood pressure (mmHg)** **T0**	93.7±13.8	94.5±10.9	t_(df=78)_ =0.16 *P*=0.795 NS
**T1**	85.6^#^±14.0	81.9^#^±10.5	t_(df=78)_ =1.34 *P*=0.183 NS
**T2**	85.1^#^±14.5	85.0±10.2	t_(df=78)_ =0.05, *P*=0.965 NS
**T3**	84.6±14.0	87.1±9.2	t_(df=78)_ =0.93, *P*=0.354 NS
**T4**	85.9±11.3	89.0±9.7	t_(df=76)_ =1.12 *P*=0.267 NS
**T5**	87.1±11.2	86.4±9.6	t_(df=64)_ =0.006 *P*=0.995 NS
**Repeated measures ANOVA** **(chi-square)** ***P***	F_(GG)_=10.043, *P*=0.000*	F=8.084 *P*=0.000*	

T0 (baseline), T1 (5 minutes after induction), T2 (5 min ERCP), T3 (15 min ERCP), T4 (30 min ERCP), and T5 (end ERCP).

*Denotes statistical significance (*P *< 0.05), while NS denotes statistical non-significance (*P* > 0.05). ^#^Statistical significance with measurement time T0

SD, standard deviation; t, independent Student’s t-test; df, degree of freedom; ERCP, endoscopic retrograde cholangiopancreatography.
